# Doppler resistive index to reflect regulation of renal vascular tone during sepsis and acute kidney injury

**DOI:** 10.1186/cc11517

**Published:** 2012-09-12

**Authors:** Antoine Dewitte, Julien Coquin, Bertrand Meyssignac, Olivier Joannès-Boyau, Catherine Fleureau, Hadrien Roze, Jean Ripoche, Gérard Janvier, Christian Combe, Alexandre Ouattara

**Affiliations:** 1 CHU de Bordeaux, Service d'Anesthésie-Réanimation II, avenue de Magellan, F-33604 Pessac, France; 2 University of Bordeaux, Bioingénierie tissulaire, U1026, 146 rue Léo-Saignat, F-33000 Bordeaux, France; 3 University of Bordeaux, Adaptation cardiovasculaire à l'ischémie, U1034, 125 avenue du Haut-Lévèque, F-33600 Pessac, France; 4 CHU de Bordeaux, Service de Néphrologie Transplantation Dialyse, place Amélie Raba-Léon, F-33000 Bordeaux, France

## Abstract

**Introduction:**

Renal resistive index (RI), determined by Doppler ultrasonography, directly reveals and quantifies modifications in renal vascular resistance. The aim of this study was to evaluate if mean arterial pressure (MAP) is determinant of renal RI in septic, critically ill patients suffering or not from acute kidney injury (AKI).

**Methods:**

This prospective observational study included 96 patients. AKI was defined according to RIFLE criteria and transient or persistent AKI according to renal recovery within 3 days.

**Results:**

Median renal RIs were 0.72 (0.68-0.75) in patients without AKI and 0.76 (0.72-0.80) in patients with AKI (*P*=0.001). RIs were 0.75 (0.72-0.79) in transient AKI and 0.77 (0.70-0.80) in persistent AKI (*P*=0.84). RI did not differ in patients given norepinephrine infusion and was not correlated with norepinephrine dose. RI was correlated with MAP (ρ= -0.47; *P*=0.002), PaO_2_/FiO_2 _ratio (ρ= -0.33; *P*=0.04) and age (ρ=0.35; *P*=0.015) only in patients without AKI.

**Conclusions:**

A poor correlation between renal RI and MAP, age, or PaO_2_/FiO_2 _ratio was found in septic and critically ill patients without AKI compared to patients with AKI. These findings suggest that determinants of RI are multiple. Renal circulatory response to sepsis estimated by Doppler ultrasonography cannot reliably be predicted simply from changes in systemic hemodynamics. As many factors influence its value, the interest in a single RI measurement at ICU admission to determine optimal MAP remains uncertain.

## Introduction

In critically ill patients, sepsis and acute kidney injury (AKI) are very common diseases and are associated with increased hospitalization and elevated in-hospital mortality rates [1,2]. AKI is a dynamic process that evolves from an early reversible condition to an established disease and leads to sustained renal impairment, cell death, and delayed renal recovery [3,4]. Consequently, prompt resuscitation of the circulation and optimal perfusion pressure are the primary therapies for critically ill patients with AKI. These methods are based principally on the appropriate management of intravenous fluid replacement and vasopressor administration under strict hemodynamic monitoring [5].

To date, the pathogenesis of septic AKI is not completely known [6]. An increase in renal vascular resistance (RVR), associated with reduced renal blood flow (RBF) and, thus, potential renal ischemia has been reported [7,8]. Conversely, some experimental studies have demonstrated that septic AKI may be associated with renal vasodilatation and increased RBF [9,10]. Renal resistive index (RI), determined by Doppler ultrasonography, allows an estimation of RVR at the bedside. Although this parameter provides interesting information, its interpretation remains difficult in clinical practice. In patients with septic shock, the norepinephrine (NE)-induced increase in mean arterial pressure (MAP) from 65 to 75 mm Hg is associated with significant decreases in RVR and RI [11]. In contrast, some authors have reported that RI was not influenced by vasoactive drug therapy [12,13]. Furthermore, a recent publication reported that increased RI was associated with persistent AKI in critically ill patients who require mechanical ventilation [12]. We tested the hypothesis that the relationship between renal RI and MAP may be influenced by renal injury. The primary objective of this study was to evaluate whether MAP is a determinant of renal RI in septic and critically ill patients who do or do not have AKI.

## Materials and methods

### Setting and patients

Approval for this non-interventional study was obtained from our institutional review board (Comité de Protection des Personnes Sud-Ouest et Outre Mer III, agreement number DC2009/43, Bordeaux, France). Because data were collected while the care of patients conformed to standard procedures currently used in our institute, authorization to waive informed consent for the study was granted. From January 2010 to February 2011, patients admitted to our 22-bed mixed medical and surgical intensive care unit (ICU) from less than 24 hours for proven or suspected infection with severe sepsis or shock according to the American College of Chest Physicians/Society of Critical Care Medicine classification [14] were prospectively included. Non-inclusion criteria were pregnancy, age of less than 18 years, known renal artery stenosis, known end-stage renal disease or chronic kidney disease defined by a glomerular filtration rate (GFR) of less than 30 mL/minute per 1.73 m^2^, angiotensin-converting enzyme inhibitor and non-steroidal anti-inflammatory treatment, cirrhosis with hepatorenal syndrome, and a major cardiogenic component in septic shock defined as a need for epinephrine or dobutamine or both. Patients presenting obstructive acute renal failures, assessed by echography, were also excluded.

### Study protocol

Each patient was studied within the first 24 hours after ICU admission and after obtaining hemodynamic stabilization defined as an MAP of greater than 60 mm Hg for more than 1 hour without any change in the rate of catecholamine infusion or fluid vascular loading. Variation in pulse pressure by arterial catheterization (radial or femoral) was used as a trigger for fluid challenge. NE was the only vasopressor used in this study. Owing to the observational nature of the study, the optimum MAP was left to the discretion of the attending physicians. Fluid balance on inclusion day was calculated by hourly recording of all intravenous fluid intakes (including colloids, crystalloids, total parenteral nutrition, or blood products) and urinary or digestive output from each patient. The ventilator respiratory settings and sedative infusion rates were unchanged for at least 1 hour before Doppler sonography. Sedation was given according to individual needs as assessed by the Behavioral Pain Scale [15] and the Sedation-Agitation Scale [16]. Renal replacement therapy was initiated only in patients in the Failure stage of Risk, Injury, Failure, Loss, and End-stage kidney disease (RIFLE).

Transthoracic echocardiography and renal echocardiography were systematically performed by intensivists certified in echocardiography. A Vivid-i ultrasound machine with a 4-MHz curved-array multifrequency transducer was used (GE Healthcare, Little Chalfont, Buckinghamshire, UK). The cardiac output by using the Doppler method at the level of the left ventricular outflow tract and renal perfusion indices were measured [17]. Intra-renal Doppler signals were obtained from two or three representative proximal interlobar arteries. The peak systolic velocity (V_max_) and the minimal diastolic velocity (V_min_) were determined by pulse wave Doppler, and the RI was calculated as (V_max _- V_min_)/V_max _[13]. The results from three consecutive measurements on both kidneys were averaged. The reproducibility of RI measurements was assessed in 20 patients by two investigators (AD and BM).

### Definitions

The Simplified Acute Physiology Score II (SAPS II) and the Sepsis-related Organ Failure Assessment score were assessed to evaluate the severity of disease of each patient at inclusion in the study. AKI was evaluated at inclusion and was defined by using the consensus definition from the Acute Dialysis Quality Initiative group, which proposes criteria for three grades of increasing severity (risk of acute renal failure, injury to the kidney, and failure of kidney function) and two outcome classes (loss of kidney function and end-stage kidney disease) (RIFLE classification). In the absence of known baseline serum creatinine (sCr) levels, the nadir of sCr after renal recovery was recorded to evaluate baseline renal function. In the absence of renal recovery, baseline sCr was estimated by using the Modification of Diet in Renal Disease (MDRD) formula. Transient AKI was defined as AKI that resolved within 3 days after inclusion by using conventional treatment in the ICU. Recovery from AKI was defined as a 50% decrease in sCr or normalization of urine output in the absence of diuretics or both. Persistent AKI was defined as persistent elevated sCr or oliguria (less than 0.5 mL/kg per hour) for at least 3 days [18-21].

### Statistical analyses

Continuous variables were presented as mean (standard deviation) and median (interquartile range) for non-Gaussian variables and were compared by the Mann-Whitney *U *test. Categorical variables were presented as number (percentage) and were compared by the Pearson chi-squared test or Fisher exact test. Comparisons between more than two groups were made by using Kruskal-Wallis one-way analysis of variance with *post hoc *analysis. Intra-observer reliability and inter-observer reliability of measurements were determined by the corresponding intraclass correlation coefficients and their 95% confidence intervals (CIs) [22]. Inter-observer measurements were compared by a Bland and Altman analysis. Correlation tests were performed by using the Spearman correlation coefficient. A logistic regression was performed to identify variables significantly associated with an RI of less than 0.75. All tests were two-sided, and a *P *value of less than 0.05 was considered statistically significant. Statistical analysis was carried out with JMP for Windows, version 6.0 (SAS Institute Inc., Cary, NC, USA).

## Results

Ninety-six patients were prospectively screened during the study period. Poor-quality images from two patients made Doppler measurements for any lobar arteries impossible. Consequently, 94 patients were included in the statistical analyses (Table 1). Doppler measurements could be obtained from only one kidney in 15 patients. In this case, the right kidney has been preferentially observed (11 patients out of 15). For the 79 patients in whom bilateral Doppler could be performed, matched *t *test comparisons of right and left RI measurements showed no significant differences: 0.73 (95% CI 0.70 to 0.76) versus 0.73 (95% CI 0.70 to 0.75). Intra-observer variation was 3.4%, and the intraclass correlation coefficient was 0.94 (95% CI 0.92 to 0.96). Inter-observer variation was 6.2%, and the intraclass correlation coefficient was 0.92 (95% CI 0.81 to −0.97). The mean bias of inter-observer measurements was 0.00 (95% CI −0.10 to 0.09). RI was not correlated with cardiovascular risk factors and was correlated only with age in patients without AKI (ρ = 0.35; *P *= 0.015). RI was higher in patients presenting with an arterial partial pressure of oxygen/fraction of inspired oxygen (PaO_2_/FiO_2_) ratio of less than 200: 0.76 (0.72 to 0.80) versus 0.72 (0.68 to 0.78) (*P *= 0.02).

**Table 1:** Characteristics of patients included in the study (n = 94)

Variables	
Male gender, mean (standard deviation)	59 (63)
Age in years, median (interquartile range)	62 (17)
Body mass index in kg/m^2^, median (interquartile range)	25 (22-28)
SAPS II at inclusion, median (interquartile range)	43 (33-59)
SOFA score at inclusion, median (interquartile range)	7 (3-9)
Mechanical ventilation, number (percentage)	60 (64)
ICU length of stay in days, mean (standard deviation)	14 (13)
Patients receiving norepinephrine, number (percentage)	47 (50)
Norepinephrine dose in μg/kg per minute, mean (standard deviation)	0.37 (0.34)
Cardiovascular risk factors, number (percentage)	
Tobacco	35 (37)
Hypertension	47 (50)
Diabetes	12 (13)
Elevated cholesterol	21 (22)
Obesity and physical inactivity	9 (10)
Origin of sepsis, number (percentage)	
Abdominal	55 (59)
Pulmonary	26 (28)
Mediastinitis	6 (6)
Catheter	2 (2)
Other	5 (5)
Diuretic use, number (percentage)	20 (21)
Acute kidney injury, number (percentage)	52 (55)
Transient	24 (25)
Persistent	28 (30)
Renal replacement therapy, number (percentage)	20 (21)
28-day all-cause mortality, number (percentage)	18 (20)

ICU, intensive care unit; SAPS II, Simplified Acute Physiology Score II; SOFA, Sepsis-related Organ Failure Assessment.

For patients with transient AKI, diagnosis was made at Risk, Injury, or Failure levels in 42%, 33%, and 25% of cases, respectively. For patients with persistent AKI, diagnosis was made at Risk, Injury, or Failure levels in 7%, 11%, and 82% of cases, respectively. Median renal RIs were 0.72 (0.68 to 0.75) in patients without AKI and 0.76 (0.72 to 0.80) in patients with AKI (*P *= 0.001) (Table 2). Median RIs were 0.75 (0.72 to 0.79) in cases of transient AKI and 0.77 (0.70 to 0.80) in cases of persistent AKI (*P *= 0.84).

**Table 2:** Characteristics of patients according to acute kidney injury

	No AKI (n = 42)	Transient AKI (n = 24)	Persistent AKI (n = 28)	*P *value
Mean arterial pressure, mm Hg	78 (67-86)	70 (65-87)	70 (65-87)	0.4
Heart rate, beats per minute	92 (78-105)	90 (83-120)	96 (79-117)	0.5
Fluid balance on inclusion day, mL	1,585 (837-2,612)^a^	1,725 (1,205-3,002)	2,650 (1,556-3,487)^b^	0.01
Norepinephrine dose, μg/kg per minute	0.18 (0.06-0.37)^a^	0.29 (0.11-0.45)^a^	0.48 (0.20-0.78)^b,c^	0.01
Cardiac index, L/minute per m²	3.1 (2.3-4.6)	3.3 (2.3-3.8)	3.1 (2.4-4.2)	1
Blood lactate, mEq/L	1.2 (0.9-2.0)^a^	1.5 (1.0-2.9)	2.4 (1.3-3.9)^b^	0.02
Hematocrit, percentage	30 (27-33)	31 (28-38)	29 (25-32)	0.3
PaO_2_/FiO_2 _ratio	240 (140-320)	240 (140-305)	180 (120-260)	0.1
Serum creatinine at inclusion, μmol/L	69 (45-81)^a,c^	160 (98-196)^a,b^	191 (134-243)^b,c^	<0.0001
FeNa, percentage	0.5 (0.2-1)^a,c^	1.5 (0.6-2.4)^b^	2.7 (0.6-6.7)^b^	0.008
FeU, percentage	28 (16-39)	36 (31-45)	31 (12-46)	0.2
Resistance index	0.72 (0.68-0.75)^a,c^	0.75 (0.72-0.79)^b^	0.77 (0.70-0.80)^b^	0.005

Results are expressed as median (interquartile range). FeNa, fractional excretion of sodium, was calculated as [(urine sodium/plasma sodium)/(urine creatinine/plasma creatinine)] × 100. FeU, fractional excretion of urea, was calculated as [(urine urea/plasma urea)/(urine creatinine/plasma creatinine)] × 100. *P *value refers to between-group comparisons (Kruskal-Wallis one-way analysis of variance with *post hoc *analysis). ^a^*P *<0.05 compared with persistent acute kidney injury (AKI) group. ^b^*P *<0.05 compared with no AKI group. ^c^*P *<0.05 compared with transient AKI group. PaO_2_/FiO_2_, arterial partial pressure of oxygen/fraction of inspired oxygen.

RI did not differ between patients who received or did not receive NE - respectively, 0.73 (0.69 to 0.78) versus 0.75 (0.68 to 0.78) (*P *= 0.94) - and was not correlated with the NE dose (ρ = -0.06; *P *= 0.69). Among patients without AKI, RIs were 0.73 (0.68 to 0.76) in patients who received NE and 0.70 (0.68 to 0.75) in patients who did not receive NE (*P *= 0.30) and were also not correlated with NE dose (ρ = 0.20; *P *= 0.43).

**Figure 1:**
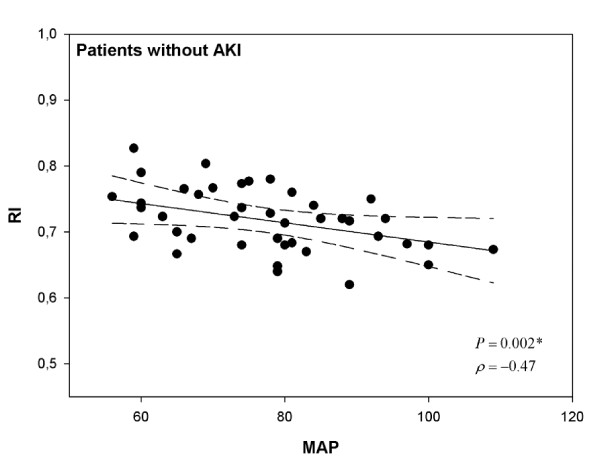
**Relationship between resistive index (RI) and mean arterial pressure (MAP) in patients without acute kidney injury (AKI)**. Correlations were assessed by using Spearman correlation coefficient.

**Figure 2:**
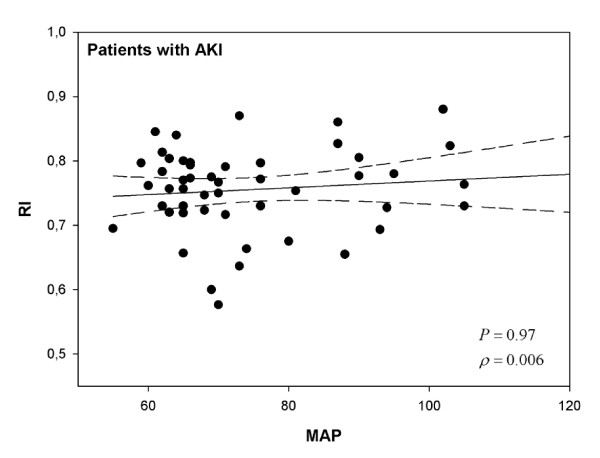
**Relationship between resistive index (RI) and mean arterial pressure (MAP) in patients with acute kidney injury (AKI)**. Correlations were assessed by using Spearman correlation coefficient.

Among patients without AKI, RI was not correlated with fluid balance on the day of inclusion (ρ = 0.21, *P *= 0.18) or with the cardiac index (ρ = -0.21; *P *= 0.37) but was significantly correlated with the MAP (ρ = -0.47; *P *= 0.002) (Figure 1) and the PaO_2_/FiO_2 _ratio (ρ = -0.33; *P *= 0.04). Among patients with AKI, RI was not correlated with fluid balance (ρ = -0.15, *P *= 0.29), the cardiac index (ρ = -0.21; *P *= 0.37), the MAP (ρ = 0.006; *P *= 0.97) (Figure 2), or the PaO_2_/FiO_2 _ratio (ρ = -0.23; *P *= 0.11). In cases of transient or persistent AKI, RI was not correlated with the MAP (ρ = 0.009, *P *= 0.99, and ρ = -0.0003, *P *= 0.99, respectively). When patients were divided into three groups according to MAP, RI was only inversely proportional to MAP in patients without AKI (*P *= 0.04) (Table 3). In contrast, in cases of AKI, RI remained elevated regardless of the MAP range. Logistic regression, using a univariate analysis, indicated that the factors that predicted an RI of greater than 0.75 in patients without AKI were the MAP (*P *= 0.02) and SAPS II (*P *= 0.002).

**Table 3:** Characteristics of patients according to mean arterial pressure

	<70 mm Hg	70 to 85 mm Hg	>85 mm Hg	*P *value
No acute kidney injury	(n = 13)	(n = 19)	(n = 10)	
Mean arterial pressure, mm Hg	63 (59-66)^a,b^	79 (74-81)^b,c^	93 (89-100)^a,c^	<0.001
Heart rate, beats per minute	88 (77-92)^a^	98 (90-115)^b,c^	90 (64-96)^a^	0.026
Fluid balance on inclusion day, mL	1,400 (865-2,735)	1,680 (990-2,560)	1,237 (699-2,376)	0.7
Norepinephrine infusion, number (percentage)	6 (46)	9 (47)	3 (30)	0.6
Norepinephrine dose, μg/kg per minute	0.20 (0.06-0.38)	0.19 (0.05-0.36)	0.17 (0.06-0.47)	0.9
Cardiac index, L/minute per m²	2.6 (1.7-3.0)	4.1 (3.1-7.8)	2.7 (1.9-5.0)	0.1
PaO_2_/FiO_2 _ratio	280 (116-310)	240 (107-302)	229 (193-395)	0.4
Resistance index	0.74 (0.70-0.78)	0.72 (0.68-0.77)	0.69 (0.67-0.72)^c^	0.04
Acute kidney injury	(n = 25)	(n = 14)	(n = 13)	
Mean arterial pressure, mm Hg	65 (62-66)^a,b^	73 (70-76)^b,c^	94 (89-104)^a,c^	<0.001
Heart rate, beats per minute	95 (83-124)	90 (82-117)	89 (79-112)	0.7
Fluid balance on inclusion day, mL	2,605 (1,303-3,767)	2,650 (2,184-3,481)	1,480 (1,105-2,400)	0.1
Norepinephrine infusion, number (percentage)	16 (64)	10 (71)	3 (23)	0.02
Norepinephrine dose, μg/kg per minute	0.20 (0.06-0.38)	0.19 (0.05-0.36)	0.17 (0.06-0.47)	0.9
Cardiac index, L/minute per m²	2.8 (2.4-3.6)	3.0 (1.8-5.2)	3.7 (3.1-5.9)	0.4
PaO_2_/FiO_2 _ratio	140 (110-270)	252 (180-310)	180 (134-270)	0.06
Resistance index	0.76 (0.73.80)	0.74 (0.66-0.78)	0.78 (0.73-0.82)	0.1

Results are expressed as median (interquartile range). *P *value refers to between-group comparisons (Kruskal-Wallis one-way analysis of variance with *post hoc *analysis). ^a^*P *<0.05 compared with mean arterial pressure (MAP) 70-85 mm Hg group. ^b^*P *<0.05 compared with MAP >85 mm Hg group. ^c^*P *<0.05 compared with MAP <70 mm Hg group. PaO_2_/FiO_2_, arterial partial pressure of oxygen/fraction of inspired oxygen.

## Discussion

In this study, renal RI was evaluated in critically ill patients with sepsis after hemodynamic stabilization. A poor correlation between RI and MAP, age, or PaO_2_/FiO_2 _ratio was found in septic and critically ill patients without AKI compared with patients with AKI. Our understanding of the pathogenesis of septic AKI is limited because currently we do not have a clinically validated method to measure RBF. In patients with renal hypoperfusion, compensatory mechanisms to maintain GFR include attenuation of afferent arteriolar vasoconstriction, afferent arteriolar dilation, efferent arteriolar constriction, and neuro-/hormonal effects to increase tubular reabsorption of fluid and maintenance of cardiac output [18]. An increase in RVR, representing renal vasoconstriction and a reduced RBF leading to renal ischemia, has repeatedly been proposed to be central to the pathogenesis of septic acute renal failure [7,8]. This paradigm has been derived from animal studies, using a variety of models, and from techniques for the measurement of RBF and for induction of sepsis without hemodynamic support [23]. However, many studies do not report decreased RBF in sepsis. Shortly after induced septic shock in ewes without significant fluid resuscitation or NE administration, Langenberg and colleagues [10] reported marked renal vasodilatation, which led to a transient increase in RBF. In parallel, sCr significantly increased after 24 hours whereas fractional excretion of sodium and fractional excretion of urea decreased in the first 48 hours (as seen in pre-renal azotemia). In human sepsis, sustained systemic vasodilatation with a high cardiac output is the dominant clinical finding during the early phase of AKI [24,25].

Determination of increased RI on the first day of septic shock is known to have predictive value for acute renal failure in septic shock [13]. Darmon and colleagues [12] recently confirmed these results in patients who required mechanical ventilation. A Doppler renal RI value of more than 0.795 also predicted persistent AKI with good sensitivity and specificity. Our study has confirmed that RI is increased in cases of AKI, particularly in cases of persistent AKI. However, we were not able to precisely distinguish transient from persistent AKI in our selected patients. We assume that hypoperfusion (pre-renal component) and injury (renal component) often occurred at the same time in injured kidneys given that each tubular injury is a continuum from volume responsiveness to necrosis. The definition of pre-renal azotemia has conceptual or diagnostic limitations or both, and a precise renal RI cutoff point to distinguish clinical entities was difficult to determine in this study.

Alterations in renal hemodynamics may be induced by multiple factors in critically ill patients. Improving regional perfusion and inducing excessive vasoconstriction must be balanced when NE therapy is required. Indeed, intra-renal infusion of NE is used to induce AKI in animals, and NE has been shown to decrease RBF in healthy volunteers. In contrast, too low a dose of NE in patients with vasodilatory shock may result in an MAP that is below the limit of renal autoregulatory capacity. Guidelines for hemodynamic support of adult patients with sepsis advocate that vasopressors be titrated to the minimum level required to provide effective organ perfusion [26]. To prevent AKI, maintaining an MAP of at least 60 to 65 mm Hg during vasodilatory shock has also been recommended [27].

Deruddre and colleagues [11] suggested that measuring renal RI could help optimize renal perfusion in critically ill patients. In that study, a significant decrease in renal RI was observed in 11 patients with septic shock when MAP was increased from 65 to 75 mm Hg by NE titration. However, measurements of renal RI remain difficult to interpret in clinical practice. Our findings confirm previous results, showing that RI is dependent on factors apart from NE infusion [12,13]. In patients with acute lung injury, low oxygenation levels have been reported to increase renal RI [28]. In our study, RI was inversely correlated with the PaO_2_/FiO_2 _ratio in patients without AKI. Recently, Darmon and colleagues [12] also found a significant negative correlation between MAP and RI in critically ill patients receiving mechanical ventilation (see Online Supplementary Material). However, in that study, MAP was not statistically different between patients without AKI and patients with transient or persistent AKI, despite an increasing RI.

Our study has reported that RI is inversely correlated with MAP in septic critically ill patients without AKI but not in patients with AKI. This may have been because of unresponsive renal vasoconstriction or renal vascular obstruction caused by a sustained kidney injury, despite hemodynamic support, which led to increased RI. On the other hand, in septic critically ill patients without AKI, an inverse correlation might reflect increased renal perfusion pressure with associated reduced activation of RVR, reflected by RI, leading to pressure-dependent renal perfusion. Sepsis or sedation could explain this modified renal vasomotor response in our patients. However, this association seems to be relatively poor. Indeed, the correlation coefficient that we found between RI and MAP means that most of the changes in RI lie somewhere other than MAP. Consequently, renal circulatory response to sepsis estimated by Doppler ultrasonography cannot be reliably predicted simply from changes in systemic hemodynamics. This finding is consistent with a recent study showing that the development of septic AKI often seems to be accompanied by an uncoupling between systemic vascular resistance and RVR [29].

The following points need to be considered when assessing the clinical relevance of our study. First, this study was purely observational as it was difficult to perform a blinded study. Second, the present study included a single-center population of patients who had developed vasodilatory shock. Patients exhibiting an important cardiogenic component of septic shock, defined by the need for epinephrine or dobutamine or both, were deliberately excluded. Third, the pressure of the renal interstitium was impossible to measure in clinical practice, and renal Doppler measurements do not reflect microcirculation vessel tone. In addition, measurement of GFR and renal oxygenation by continuous renal vein thermodilution was not conducted, as we were unable to use invasive hemodynamic monitoring in this observational study. No patient included in this study presented clinical intra-abdominal hypertension, but this was not assessed by intravesical measurement. Other confounding factors not identified in this study also may have influenced our results. Finally, the subjective part of renal ultrasound and the precision of measurements, assessed by the inter-observer variation in comparison with the Doppler measurement differences, are the main limitations when determining renal RI.

Although future AKI studies need to determine the impact of early recognition of pressure-dependent renal perfusion on renal recovery, the timing of volume replacement to facilitate early restoration of kidney perfusion and to optimize vasopressor infusion dose can be an important determinant in preventing the progression of AKI and facilitating recovery. Estimating renal circulatory response to sepsis by Doppler ultrasonography can be hazardous because many factors influence RI value. Increased RI may be associated with low renal perfusion pressure or low oxygenation levels in patients without AKI but may also be associated with sustained renal injury. Thus, its value in septic critically ill patients in the ICU should be interpreted carefully before concluding that there is renal injury or estimating optimal MAP.

## Conclusions

A poor correlation between renal RI and MAP found only in septic and critically ill patients without AKI suggests that determinants of RI are numerous. Consequently, renal circulatory response to sepsis estimated by Doppler ultrasonography cannot reliably be predicted simply from changes in systemic hemodynamics. The value of a single RI measurement at ICU admission to determine optimal MAP remains uncertain.

## Key messages

• RI, measured by Doppler ultrasonography, is correlated to MAP, age, and PaO_2_/FiO_2 _ratio only in septic and critically ill patients without AKI.

• RI was increased in cases of AKI, but theses correlations were abolished.

• RI did not differ between patients who received or did not receive NE and was not correlated with the NE dose.

• This correlation between RI and MAP in septic and critically ill patients without AKI is poor, suggesting that the determinants of RI are numerous.

• A single RI measurement at admission of septic critically ill patients to predict persistent AKI or to determine optimal MAP seems insufficient.

## Abbreviations

AKI: acute kidney injury; CI: confidence interval; GFR: glomerular filtration rate; ICU: intensive care unit; MAP: mean arterial pressure; NE: norepinephrine; PaO_2_/FiO_2_: arterial partial pressure of oxygen/fraction of inspired oxygen; RBF: renal blood flow; RI: resistive index; RVR: renal vascular resistance; SAPS II: Simplified Acute Physiology Score II; sCr: serum creatinine.

## Competing interests

The authors declare that they have no competing interests.

## Authors' contributions

AD conceived, designed, and coordinated the study. BM and JC helped to collect the clinical data. AO helped to carry out the statistical analysis. OJ-B, CF, HR, JR, GJ, CC and AO helped to critically revise the manuscript. All authors read and approved the final manuscript.
